# Reduced Seasonal Coronavirus Antibody Responses in Children Following COVID-19 Mitigation Measures, The Netherlands

**DOI:** 10.3390/v15010212

**Published:** 2023-01-12

**Authors:** Reina S. Sikkema, Erwin de Bruin, Christian Ramakers, Robbert Bentvelsen, Wentao Li, Berend-Jan Bosch, Brenda Westerhuis, Bart Haagmans, Marion P. G. Koopmans, Pieter L. A. Fraaij

**Affiliations:** 1 Viroscience, Erasmus MC, 3015 Rotterdam, The Netherlands; 2 Clinical Chemistry, Erasmus MC, 3015 Rotterdam, The Netherlands; 3 Microvida Laboratory for Microbiology, Amphia Hospital, 4818 Breda, The Netherlands; 4 Department of Medical Microbiology, Leiden University Medical Center, 2333 Leiden, The Netherlands; 5 Infectious Diseases & Immunology, Faculty of Veterinary Medicine, Utrecht University, 3584 Utrecht, The Netherlands; 6 Pediatrics, Erasmus MC-Sophia Children’s Hospital, 3015 Rotterdam, The Netherlands

**Keywords:** children, seasonal coronavirus, COVID-19, serosurvey, antibodies

## Abstract

SARS-CoV-2 prevention and control measures did not only impact SARS-CoV-2 circulation, but also the timing and prevalence of other seasonal respiratory viruses. Especially in children, information on exposure and infections to seasonal coronaviruses as well as SARS-CoV-2 in the first year of the pandemic is largely lacking. Therefore, we set up a one-year serological survey in a large tertiary hospital in the Netherlands. We show that seasonal coronavirus seroprevalence significantly decreased in 2021 in children less than one year, most likely due to COVID-19 control measures. The SARS-CoV-2 seroprevalence in children and adolescents increased from 0.4% to 11.3%, the highest in adolescents. This implies higher exposure rates in adolescents as compared to the general population (>18 years old). It is clear that there have been significant changes in the circulation and subsequent immunity against most respiratory pathogens as a result of the mitigation measures. The implications on shorter as well as longer term are still largely unknown, but the impact of the SARS-CoV-2 pandemic and subsequent control measures will continue to affect the dynamics of other pathogens.

## 1. Introduction

According to the World Health Organisation, over half a billion individuals have been infected with the SARS-CoV-2 virus since the beginning of 2020, resulting in over 6 million deaths. As not all cases were documented this is likely an underestimation of the true impact of the SARS-CoV-2 virus pandemic (https://www.who.int/data/stories/the-true-death-toll-of-covid-19-estimating-global-excess-mortality; accessed on 6 December 2022). The ferocity of this pandemic resulted in governments implementing strict prevention and control measures including nationwide lockdowns, work-from-home instructions, physical distancing, and school closures. Although the impact of these measures is still under scrutiny, it is apparent that they did not only impact SARS-CoV-2, but also other circulating seasonal viruses [1,2]. This was for instance shown by the fact that in some regions seasonal influenza virus and respiratory syncytial virus (RSV) outbreaks were less profound and no longer occurred in autumn and winter or even at all [3,4]. For some ubiquitously circulating viruses, such as the seasonal human coronaviruses, not a lot is known about the exposure and immunity, especially in the first year of the pandemic.

There are four different seasonal human coronaviruses, belonging to the genera Alphacoronavirus and Betacoronavirus: 229E, NL63, OC43, and HKU1. Most seasonal corona infections are reported during childhood or adolescence [5]. The majority of children seroconvert early in childhood and are re-infected multiple times [6,7]. They usually cause mild respiratory symptoms, also known as the common cold, and are estimated to be responsible for 15–30% of all respiratory infections [1].

In accordance with human seasonal coronaviruses, most children and adolescents develop only mild disease or even stay asymptomatic after infection with SARS-CoV-2. Severe disease is uncommon and usually linked to underlying medical conditions [8]. However, a rare post-infectious complication is a multisystem inflammatory syndrome (MIS), that can affect children (MIC-C). Interestingly, the number of cases of MIS-C appears to decline during the course of the pandemic, which is thought to be related to an increase in immune protection with the increasing number of children infected with SARS-CoV-2 [9].

In the first year of the COVID-19 pandemic, there was a restricted testing policy mainly focusing on severely symptomatic individuals [10]. Moreover, in the Netherlands polymerase chain reaction (PCR) of symptomatic children below the age of 12 was only implemented structurally in 2021. Since children and adolescents rarely develop severe disease, little is known if and how SARS-CoV-2 spread in the lower age groups in the first year of the pandemic.

To better understand the impact of SARS-CoV-2 and associated mitigation measures on seasonal coronavirus infections in children and adolescents in the Netherlands in the first year of the pandemic and to assess the SARS-CoV-2 infection rate in children, we set up a one-year serological survey, using plasma samples submitted for clinical chemistry analyses in a large tertiary hospital in the Netherlands. Our data provide insight into the effects of the COVID mitigation measures on seroconversion to seasonal coronaviruses as well as the number of infections with SARS-CoV-2 in the different age groups in the first year of the pandemic in The Netherlands.

## 2. Material and Methods

### 2.1. Sample Collection

Leftover lithium-heparin plasma samples submitted for general 24/7 clinical chemistry analyses were collected at the Erasmus medical center. Samples from (suspect) COVID-19 patients were automatically excluded regardless of the result. The collection of plasma samples in 2020 was done over a 5-day period at two time points, starting 9 March and 10 April. The 2021 samples were collected from 22 January until 4 March. Samples were retrieved after 4 to 7 days of cold storage (4C), depending on storage capacity. Only age category information was collected: 6–12 months; 1–2 year; 2–5 year; 5–10 year; 10–18 year. Samples from children aged <6 months were excluded as antibodies in this age bracket mostly are derived from maternal transplacental transfer.

### 2.2. Protein Microarray

All sera were tested against 13 different antigens from the 4 seasonal coronaviruses and pandemic coronavirus (OC43, 229E, NL63, HKU1, and SARS-CoV-2), that were printed using a non-contact printer (sciFlexarrayer SX, Scienion, Berlin, Germany) on glass microscope slides coated with 64 pads of nitrocellulose. Recombinant spike proteins of the S1 subunit (all coronavirus subtypes) or the S ectodomain (all except 229E) were expressed in HEK293 cells as described before [11]. Nucleocapsid proteins were produced in Escherichia coli (for OC43, 229E, NL63, and HKU1; Medix Biochemica, Espoo, Finland) or baculovirus and insect cells (SARS; Sino Biological, Beijing, China). 

Slides were incubated with serum for 1 h in a single 1 in 20 dilution in incubation buffer (Blocker blotto, Thermo Fisher Scientific Inc., Rockford, IL, USA, containing 0.1 percent Surfact Amps, Thermo Fisher Scientific Inc.). After serum incubation and washing, slides were incubated with goat anti-human IgG (Fab-specific) conjugated to Alexa Fluor 647 (Jackson Immunoresearch Laboratories Inc., West Grove, PA, USA). Single-spot median fluorescent signals using per-spot background correction were measured using a Powerscanner (Tecan Group Ltd., Mannedorf, Switzerland).

### 2.3. Data Analysis

**Figure 1:**
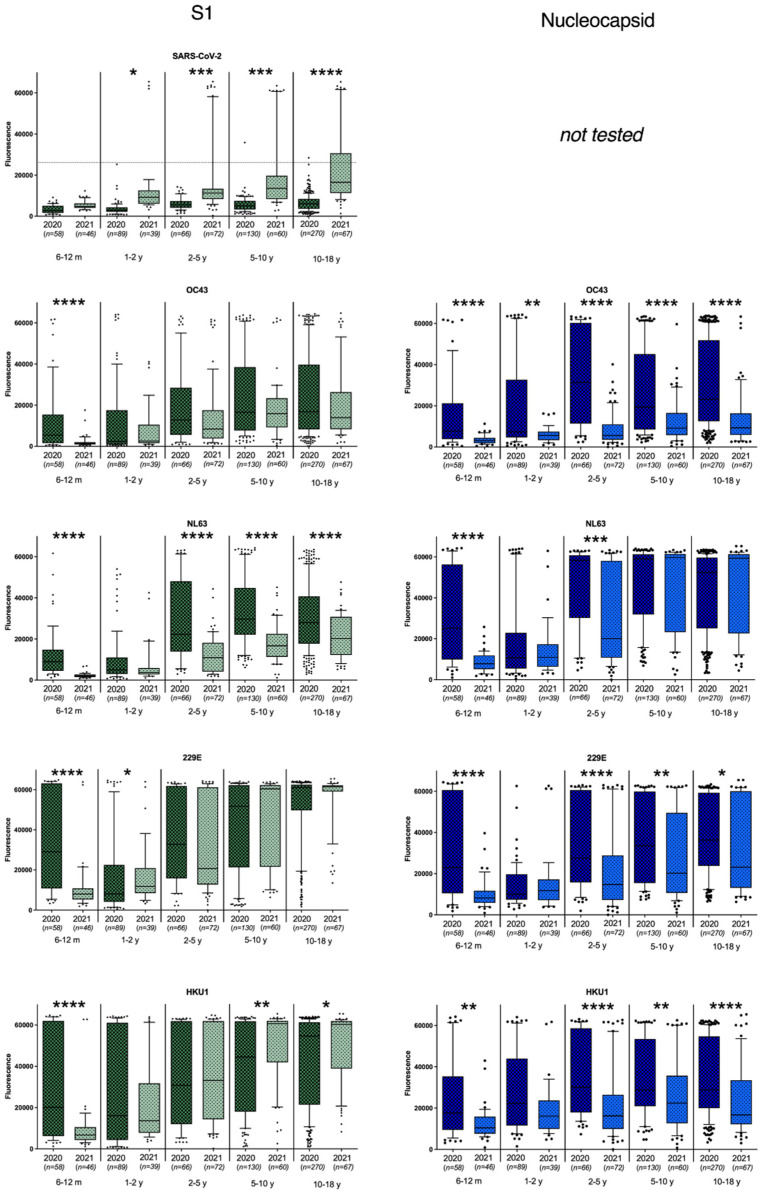
Antibody binding to S1 and nucleocapsid antigens of SARS-CoV-2, OC43, HKU1, 229E, and NL63 seasonal coronaviruses. For SARS-CoV-2 antigens, the calculated cutoff for positivity is indicated. * represents *p* < 0.05; ** represents *p* < 0.01, *** represents *p* < 0.001, **** represents *p* < 0.0001.

Fluorescence values were determined using Imagene 8.0 software (Biodiscovery, El Segundo, CA, USA). To define positivity for SARS-CoV-2, cut-offs were calculated for SARS-CoV-2 antigens by ROC-curves (GraphPad Prism, version 9.0.0, San Diego, CA, USA) using a panel of 60 sera collected before 2020 from seasonal CoV patients and a panel of confirmed positive COVID-19 patients. The cutoff is indicated in Figure 1. Samples were considered to be positive if antibody binding was detected against all SARS-CoV-2 antigen signals (NP, S1, and spike ectodomain in 2020, S1 and spike ectodomain in 2021). No cutoffs were determined for the seasonal coronaviruses. Figures were made using GraphPad Prism (version 9.0.0). Statistical differences between groups were calculated using the Mann–Whitney test (seasonal coronaviruses-sCoVs) and Fisher’s exact test (SARS-CoV-2).

## 3. Results

In total, 613 plasma samples from children between 6 months and 18 years old were collected during the first period (March–April 2020) and 285 plasma samples of the same age groups were collected one year later. Antibodies binding to antigens from seasonal human coronaviruses were present from the age of 6 months (Figure 1). The sCoV antibody reactivity was lowest in 6–24-month-old children and increased from age groups 2–5 years old to 18 years old. When comparing early 2020 and early 2021, antibody binding to S1 of all seasonal coronaviruses showed a highly significant decrease in the age group 6m–1y (Figure 1). This was confirmed by reactivity against the S ectodomain and nucleoprotein (Figure 1 and Figure S1), pointing towards a decreased exposure to sCoV within this age group. In higher age groups (>2 year), a significant decrease in antibodies was also observed against all nucleocapsid proteins (*p* < 0.01), with the exception of NL63, where a decrease was only seen in the 2–5 years age group. This pattern could partly be confirmed when looking at the antibody binding of the S1 and full spike antigen (Figure 1 and Figure S1). Notably, for HKU1 there seems to be a slight increase in antibodies binding to HKU1 S1 in children over 5 years, which could not be seen in the other HKU1 antigen responses. 

The SARS-CoV-2 antibody positivity rates were also compared between the beginning of the outbreak in the Netherlands and one year later. In April 2020, one adolescent (1/613; 0.4%) tested positive for SARS-CoV-2 antibodies while one year later, the overall seropositivity increased to 11.3% (32/284) (Figure 1; Table 1). The seropositivity rate increased with age, with still no positives detected in children below the age of one year and a maximum of 20.9% positives in adolescents (10–18 year).

## 4. Discussion

Here we show a significant decrease in antibody responses against all seasonal coronaviruses in children, specifically those aged between 6 months and 1 year, between early 2020 and 2021. In the same time period, the overall number of SARS-CoV-2 seropositive children and adolescents in the Netherlands increased from 0.4% in April 2020 to 11.3% in February 2021. The SARS-CoV-2 seroprevalence increased with age, with the highest seroprevalence in adolescents.

**Figure 2:**
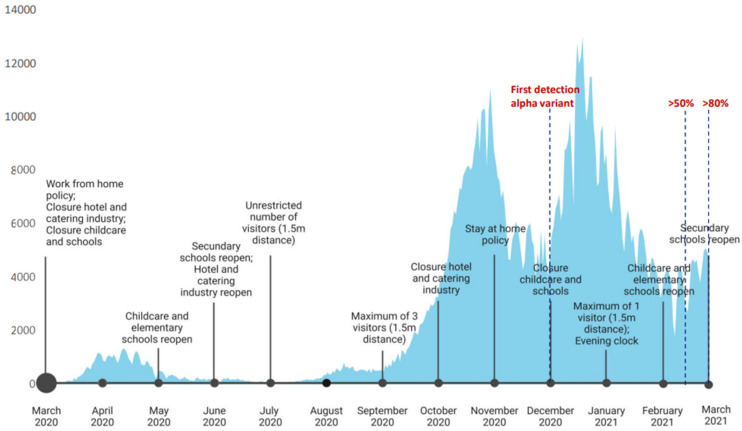
COVID-19 human cases, SARS-CoV-2 variants, and mitigation measures in the Netherlands, March 2020–March 2021. Data were obtained from the ECDC dataset on the daily number of newly reported COVID-19 cases and deaths by EU/EEA country (https://www.ecdc.europa.eu/en/publications-data/data-daily-new-cases-covid-19-eueea-country accessed on 2 January 2023) and RIVM SARS-CoV-2 variant surveillance data (https://www.rivm.nl/en/coronavirus-covid-19/virus/variants, accessed on 2 January 2023).

The decrease in sCoV antibody responses as shown for children under 1 year old and to a lesser extent also for most older age groups, can be the result of the imposed lockdown measures, including daycare closures (Figure 2). Especially, the absence of exposure to infected peers at daycare facilities during the second lockdown (December 2020 to February 2021) could have resulted in the absence of sCoV antibodies in many children in the youngest age group. The decline or absence of sCoV antibodies was less apparent for the older children, possibly explained by sCoV exposure in the years shortly before the pandemic. Moreover, since schools and daycare were not closed continuously during the epidemic in the Netherlands, older children were likely exposed to seasonal coronaviruses to some extent. Still, there was also a trend toward decreasing antibodies against sCoVs over time in the older age groups, especially when looking at antibodies directed against the nucleocapsid protein (NP). 

Most differences between antibody binding to the spike protein and the nucleocapsid protein are in agreement with previous literature describing faster decay of anti-nucleocapsid antibodies as compared to antibodies binding to the spike region [12]. Therefore, the most rapid decline is expected to be seen in NP responses, after the absence of re-exposure to sCoVs, as well as SARS-CoV-2. In general, antibodies against sCoVs wane relatively rapidly, with antibody levels below baseline in the large majority of patients one year after infection. High antibody prevalence in adults is thought to be the result of frequent reinfections [13,14] Although NP can serve as an early indicator for waning immunity, some of the nucleocapsid signals may have been influenced by cross-reactivity within coronavirus genera [15]. However, the overall decrease in antibodies across sCoV species still supports a general decrease in antibodies against all sCoVs. 

There are only a few publications that compare the SARS-CoV-2 (sero)prevalence in children under 18 years with other age groups. Although not all see a similar linear correlation between SARS-CoV-2 antibodies and age, most do see an increased exposure in adolescents as compared to children below the age of 10 years [16,17,18]. Here, we see that the number of SARS-CoV-2 positives in the age group 10–18 years even exceeded those found in the national adult blood donor screening in the same time period (20.9% vs. 14.7%) [19]. This could be due to the re-opening of secondary schools in September 2020, while older age groups (>18 years) were better able to limit their number of social contacts and continued working from home where possible (https://www.rijksoverheid.nl/actueel/nieuws/2020/08/31/alle-scholen-weer-open; accessed on 3 October 2022). 

Our findings show that lockdown measures have an impact on population immunity against common respiratory viruses in young children. This may have influenced the increase and changes in the dynamics of several viruses after measures were relaxed. For example, RSV showed a large off-season peak, including a clear increase in hospitalizations [20,21]. Moreover, other respiratory viruses, such as seasonal coronaviruses and parainfluenza virus clearly increased after SARS-CoV-2 containment measures were relaxed [22]. For the season to come our data suggest that it is not unlikely that the young will catalyze large outbreaks, as the group of immunologically “naïve” children to a certain pathogen is larger than before the lockdown period. As such, there are fears of the effects of decreased immunity and circulation in the past 2.5 years. Experts warn against a possible aggravated influenza epidemic this season, with possibly severe consequences for the very young and elderly (https://www.npr.org/sections/health-shots/2022/09/23/1124311571/flu-season-2022-covid-twindemic accessed on 2 January 2023; [23]). 

It is clear that there have been significant changes in the circulation and subsequent immunity against most respiratory pathogens as a result of the mitigation measures. The implications on shorter as well as longer term are still largely unknown, but the impact of the SARS-CoV-2 pandemic and subsequent control measures will continue to affect the dynamics of other pathogens. This means that vigilance and continued monitoring of human respiratory infections is warranted [24] Understanding current viral circulation and immunity can serve as early warning for public health institutes and clinicians and helps guide prevention and mitigation strategies.

## Figures and Tables

**Table 1:** SARS-CoV-2 seropositivity in children and adolescents, 2020–2021.

	March 2020			April 2020			January–March 2021		
	Number Positive	Number Samples	% Positive	Number Positive	Number Samples	% Positive	Number Positive	Number Samples	% Positive
6–12 months	0	41	0.0	0	17	0.0	0	46	0.0
1–2 years	0	75	0.0	0	14	0.0	3	39	7.7
2–5 years	0	42	0.0	0	24	0.0	8	72	11.1
5–10 years	0	96	0.0	0	34	0.0	7	60	11.7
10–18 years	0	131	0.0	1	139	0.7	14	67	20.9
Total	0	385	0	1	228	0.4	32	284	11.3

## Data Availability

The datasets generated during the current study are available from the corresponding author upon reasonable request.

## Supplementary Materials

The following supporting information can be downloaded at: https://www.mdpi.com/article/10.3390/v15010212/s1, Figure S1: Antibody binding to S-ectodomain antigens of SARS-CoV-2, OC43, HKU1 and NL63 seasonal coronaviruses as measured with the Protein Microarray.
