# Supplementary material for: Reduced Seasonal Coronavirus Antibody Responses in Children Following COVID-19 Mitigation Measures, The Netherlands
Source: Viruses. 2023 Jan 12;15(1):212. doi: 10.3390/v15010212 (PMC9862716; doi:10.3390/v15010212)
Supplement: Supplementary file 1 [file viruses-15-00212-s001.zip › viruses-2113861-supplementary.pdf]

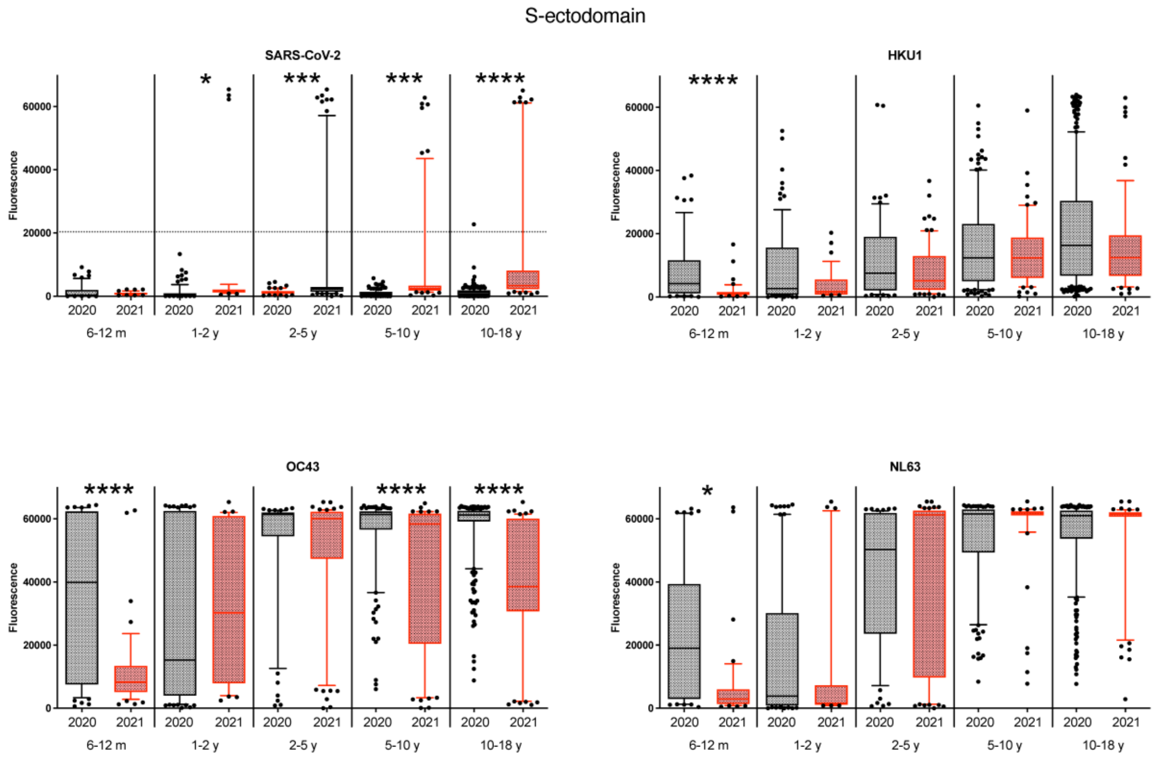

Figure S1. Antibody binding to S-ectodomain antigens of SARS-CoV-2, OC43, HKU1 and NL63 seasonal coronaviruses as measured with the ProteinMicroarray.
